# Predicting COVID-19 Outcomes: Machine Learning Predictions Across Diverse Datasets

**DOI:** 10.7759/cureus.50932

**Published:** 2023-12-22

**Authors:** Kemal Panç, Nur Hürsoy, Mustafa Başaran, Mümin Murat Yazici, Esat Kaba, Ercan Nalbant, Hasan Gündoğdu, Enes Gürün

**Affiliations:** 1 Radiology, Recep Tayyip Erdoğan Education and Research Hospital, Rize, TUR; 2 Emergency Medicine, Recep Tayyip Erdoğan Education and Research Hospital, Rize, TUR; 3 Emergency Medicine, Rize State Hospital, Rize, TUR; 4 Radiology, Samsun University, Samsun, TUR

**Keywords:** pulmonary artery diameters, lung parenchyma score, gradient boosting, artificial intelligence, covid-19

## Abstract

Background

The COVID-19 infection has spread rapidly since its emergence and has affected a large part of the global population. With the increasing number of cases, researchers are trying to predict the prognosis of patients by using different data with artificial intelligence methods such as machine learning (ML). In this study, we aimed to predict mortality risk in COVID-19 patients using ML algorithms with different datasets.

Methodology

In this retrospective study, we evaluated the fever, oxygen saturation, laboratory results, thorax computed tomography (CT) findings, and comorbid diseases at admission to the hospital of 404 patients whose diagnosis was confirmed by the reverse transcription polymerase chain reaction test. Different datasets were created by combining the data. The Synthetic Minority Oversampling Technique was used to reduce the imbalance in the dataset. K-nearest neighbors, support vector machine, stochastic gradient descent, random forest, neural network, naive Bayes, logistic regression, gradient boosting, XGBoost, and AdaBoost models were used to create the ML algorithm, and the accuracy rates of mortality prediction were compared.

Results

When the dataset was created with CT parenchyma score, pulmonary artery and inferior vena cava diameters, and laboratory results, mortality was predicted with an accuracy of 98.4% with the gradient boosting model.

Conclusions

The study demonstrates that patient prognosis can be accurately predicted using simple measurements from thorax CT scans and laboratory findings.

## Introduction

The coronavirus discovered in Wuhan, China, in December 2019, was named COVID-19 by the World Health Organization. Since its emergence, it has rapidly spread worldwide and resulted in a pandemic. As of the time of writing, the number of worldwide cases has surpassed 659 million, and the number of deaths has exceeded 6.6 million, according to data from the World Health Organization [1]. Symptoms of this ailment include fever, dry cough, muscle pain, anosmia, and gastrointestinal system complaints. The majority of symptoms are mild, however, certain patients may suffer from severe complications, which can result in death.

The increasing cases have had a critical impact on healthcare research, leading to the development of new methods to forecast patient prognosis. Predicting the mortality of COVID-19 patients is essential in recognizing individuals at a higher risk of severe illness and providing the appropriate care and interventions. Numerous studies have examined risk factors linked to negative consequences and mortality among COVID-19 patients. A systematic review and meta-analysis scrutinized the risk factors of poor outcomes in hospitalized COVID-19 patients and discovered that advanced age, male sex, obesity, diabetes, and cancer were strongly related to increased mortality risk [2]. Another study investigated the effect of asthma on mortality rates in COVID-19 patients and discovered a correlation between a history of asthma and increased mortality [3]. Along with co-existing conditions, laboratory measures have also been evaluated as prognostic markers for COVID-19 mortality. A study demonstrated the predictive potential of a diagnostic rule involving lactate dehydrogenase, high-sensitivity C-reactive protein, and lymphocytes [4]. A recent study found a correlation between the mortality risk in COVID-19 patients and their peripheral lymphocyte count [5].

Machine learning (ML) is a branch of artificial intelligence that can learn from examples and continues to evolve [6]. In the field of ML, different models have their strengths and weaknesses. The K-nearest neighbors (kNN) method is simple but can be computationally expensive. Support vector machines (SVM) are effective in high-dimensional spaces but consume a higher memory footprint. Stochastic gradient descent (SGD) is effective but sensitive to scaling. Random forests (RFs) are robust but comparatively less interpretable. Neural networks (NNs) are powerful but require a lot of data and risk overfitting. Naive Bayes is simple and quick but assumes feature independence. Logistic regression (LR) is interpretable but assumes linear relationships. Gradient boosting (GB) builds strong but is susceptible to overfitting. XGBoost improves upon this but requires careful tuning. AdaBoost combines weak models but can be sensitive to noise. Choosing the right model depends on the task at hand, with trade-offs in simplicity, interpretability, and computational efficiency. GB is an ensemble learning approach that sequentially trains weak models, typically decision trees, on residuals from the previous model. By incorporating new model predictions, this technique updates predictions iteratively, augmenting the model’s predictive capacity. Utilizing multiple weak models, this approach constructs a robust predictive model that delivers improved performance [7].

Several recent studies have focussed on using ML algorithms to predict mortality risk in COVID-19 patients [8,9]. The contribution of comorbid factors, laboratory tests, and thorax computed tomography (CT) findings to predict mortality has not been adequately investigated in the literature. In this study, we aimed to predict the mortality risk in COVID-19 patients using ML algorithms with different datasets and reveal the most effectively usable data in the pandemic.

## Materials and methods

This study was approved by the local ethics committee on September 8, 2022. Data from 404 patients were analyzed. Inclusion criteria included a thorax CT scan and laboratory tests at the time of admission to the hospital and confirmation of the diagnosis by reverse transcription polymerase chain reaction test. Exclusion criteria comprised incomplete patient files, failure to include the whole lung on thorax CT imaging, and inability to identify parenchymal findings due to respiratory artifacts. According to these criteria, 22 of 426 patients were excluded, and 404 patients were included in the study.

Age, presence of comorbidities (diabetes mellitus (DM), chronic renal failure (CRF), coronary artery disease (CAD), chronic obstructive pulmonary disease (COPD), malignancy, cerebrovascular disease (CVD) and Alzheimer’s disease), fever, and oxygen saturation at presentation were noted to create the dataset. Results of six laboratory tests including white blood cell (WBC), neutrophil (NE), lymphocyte (LE), neutrophil/lymphocyte ratio, C-reactive protein (CRP), fibrinogen, and D-dimer were added. Mortality data were recorded during the patients’ hospital stay.

The clinical status of the patients at admission was classified as mild, moderate, severe, and critical according to the seventh version of the Novel Coronavirus Pneumonia Diagnosis and Treatment Guideline published by the National Health Commission of China [10].

**Figure 1:**
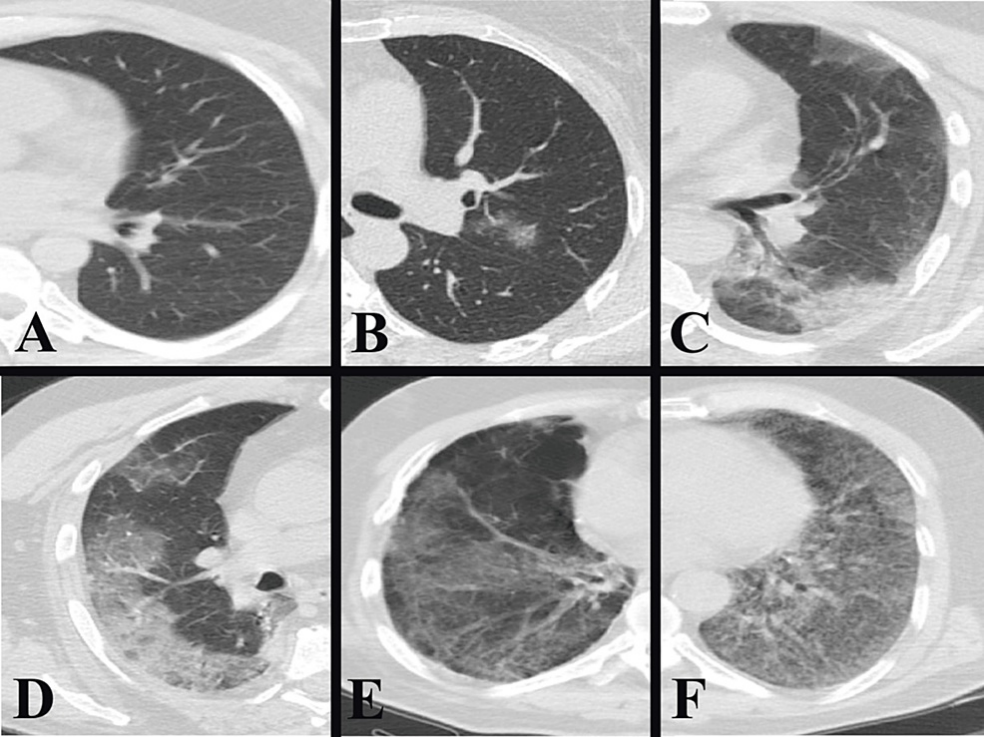
Thorax CT parenchyma scoring. Absence of parenchymal involvement score 0 (A), parenchymal involvement 0-5% score 1 (B), 5-25% score 2 (C), 25-50% score 3 (D), 50-75% score 4 (E), and 75-100% score 5 (F).

Thorax CT scans were performed using 16-slice Toshiba Alexion Advance (Alexion 16, Toshiba Medical Systems, Japan). Measurements and parenchyma scoring were made on 1 mm thin-section axial plane images. The evaluation was done by two radiologists with four and 10 years of experience. The involvement of each lung parenchyma was scored visually independently of patient information on a total of 10 points, with 1 point between 0-5%, 2 points between 5-25, 3 points between 25-50, 4 points between 50-75, and 5 points between 75-100 (Figure 1).

**Figure 2:**
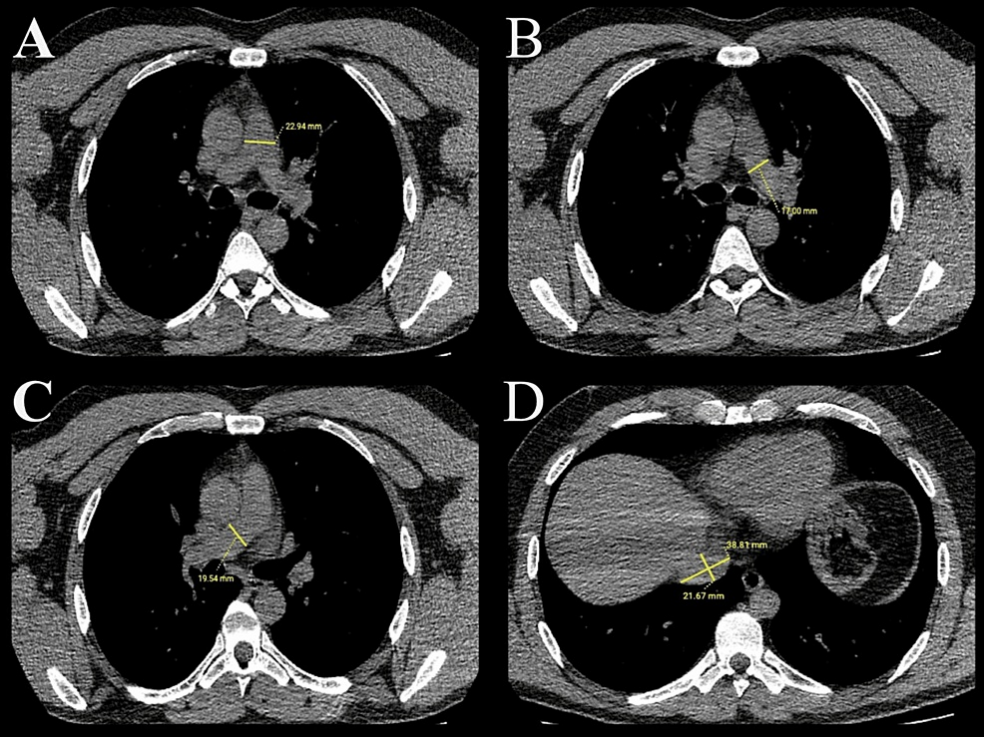
Measuring the diameters of vascular structures. In axial non-contrast CT scans, A shows the measurement of the main pulmonary artery diameter. B shows the measurement of the left pulmonary artery diameter. C shows the measurement of the right pulmonary artery diameter. D shows the measurement of the transverse and anteroposterior inferior vena cava diameter.

In addition, the presence of ground-glass opacity, consolidation, pleural effusion, halo, and reverse halo findings were recorded. The diameter of the main pulmonary artery (m-PA) was measured from the bifurcation level. The diameters of the right (r-PA) and left pulmonary arteries (l-PA) were measured from the widest diameter after the bifurcation. For the dimensions of the inferior vena cava (IVC), measurements were made in two planes from its largest diameter after exiting the liver and before pouring into the right atrium (Figure 2).

Datasets were created with different combinations of patient data. The first dataset was created using CT parenchyma score and m-PA, r-PA, l-PA, and IVC diameters. The second dataset included CT parenchyma score; m-PA, r-PA, l-PA, IVC diameters; and parenchymal findings. The third dataset included CT parenchyma score; m-PA, r-PA, l-PA, IVC diameters; and laboratory parameters, and the fourth dataset included CT parenchyma score; m-PA, r-PA, l-PA, IVC diameters; laboratory parameters; comorbid disease; age; fever; and SpO_2_.

Orange data mining software v3.34 was used to create the ML algorithm. In the first stage, patients with missing data were cleared. Numerical data were normalized between 0 and 1 to obtain a common scale. There was an imbalance of distribution between the groups in the dataset. The mortality group comprised 15.1% of the dataset. To reduce the imbalance, the Synthetic Minority Oversampling Technique (SMOTE) was used, and the class distribution in the dataset was balanced at 50%-50%.

The dataset was divided into training and test sets with a fixed ratio of 80% and 20%. The training set was divided into 10 subgroups with the 10-fold cross-validation method, trained with nine different subgroups each time, and tested with one subgroup. This process was repeated 10 times, and the validation score was obtained by taking the average of the results. After the models were trained with the 10-fold cross-validation method, they were tested with the test set (Table 1).

**Table 1:** Data handling scheme. SMOTE: Synthetic Minority Oversampling Technique

Data preprocessing	Data sampling	Models
Remove missing values	Train data (80%)	k-nearest neighbors
		Support vector machine
		Logistic regression
Normalization	10-fold cross-validation	Random forest
		Stochastic gradient descent
		Adaboost
		Naive Bayes
SMOTE	Test data (20%)	Neural network
		Gradient boosting
		XGboost

To avoid overfitting and underfitting, the acceptable gap between validation and test results, although not a standard value, was set at 3%. The accuracy of the models was evaluated by the area under the curve (AUC).

kNN, SVM, SGD, RF, NN, naive Bayes, LR, GB, XGBoost, and AdaBoost models were used to create the ML algorithm. Algorithm parameters are shown in Table 2.

**Table 2:** Parameter settings of machine learning algorithms

Machine learning algorithms/Parameters	Parameter value
k-Nearest neighbors	
Number of neighbors	5
Metrics	Eucledian
Weight	Uniform
Support vector machine	
Kernel	Sigmoid
Iteration limit	100
Random forest	
Number of trees	20
Minimum limit for split	5
Stochastic gradient descent	
Loss function	Hinge
Regularization	Lasso (L1)
Learning rate	Constant
Initial learning rate	0.01
Neural network	
Neurons in hidden layers	100
Activation	Logistic
Solver	SGD
Maximum number of iterations	200
Naive Bayes	
Prior probability estimation	Relative frequency
Logistic regression	
Regularization	Lasso (L1)
Adaptive boosting	
Number of estimators	50
Classification algorithm	SAMME.R
Regression loss function	Linear
Learning rate	0.1
Gradient boosting	
Number of trees	100
Learning rate	0.1
Fraction of training instances	1
XGBoost	
Number of trees	100
Learning rate	0.1
Fraction of training instances	1
Regularization lambda	1

All analyses were performed with the help of the Jamovi v1.6 Statistics package (Jamovi Project Computer Software, Version 1.6. Sydney, Australia). In our study, type 1 error was accepted as 5% for all comparisons. Continuous variables were defined as mean and standard deviation (SD), and categorical variables were defined by frequency and interquartile range (IQR). The Shapiro-Wilk test was used to determine whether the data were normally distributed. In the comparison of continuous variables, groups with normal distribution were compared with the t-test, and those with non-normal distribution were compared with the Mann-Whitney U test. Statistical significance was accepted as p<0.05.

## Results

A total of 404 patients were included in the study. Survival was achieved in 343 patients, while mortality occurred in 61 patients. The mean age of the patients with a mortal course was 74, and the mean age of the patients who survived was 58. The male/female ratio of the patients was 1.06, but the male/female ratio was 2.2 in patients who had a mortal course.

There were 250 patients with mild clinical status, 118 patients with moderate, and 36 patients with severe clinical status at admission to the hospital. Mortality was 1.6% in patients with mild clinical presentation, 26.2% in moderate patients, and 72.2% in severe patients (Table 3).

**Table 3:** Gender, age, and disease severity. Note: Normally distributed data are shown as mean ± SD (minimum-maximum), and non-normally distributed data as median (IQR 25-75). IQR: interquartile range

		All patients (N = 404)	Survival (N = 343)	Mortality (N = 61)
Gender, n (%)	Male n (%)	196 (48.5%)	154 (38.1%)	42 (10.4%)
	Female n (%)	208 (51.5%)	189 (46.8%)	19 (4.7%)
Age (years), n (%)		62 (IQR 49–72)	60 (IQR 47–70)	74 (IQR 68–81)
Severity of disease, n (%)	Mild	250 (61.9%)	246 (60.9%)	4 (1.0%)
	Moderate	118 (29.2%)	87 (21.5%)	31 (7.7%)
	Severe	36 (8.9%)	10 (2.5%)	26 (6.4%)

There was a significant correlation between age, oxygen saturation, and mortality risk (p < 0.001). The relationship between fever and mortality was not significant.

There was a significant correlation between diameters of the m-PA (p < 0.001), r-PA (p < 0.001), l-PA (p < 0.001), transverse diameter of the IVC (p = 0.04), and CT parenchyma score (p < 0.001) with mortality. A significant correlation was found between mortality and the presence of ground-glass density (p = 0.01) and pleural effusion (p = 0.001). However, mortality was not significantly correlated with consolidation, halo, and reverse halo findings.

A significant correlation was found between mortality and comorbidities such as HT (p = 0.004), CAD (p < 0.001), DM (p = 0.016), history of CVD (p < 0.001), and history of malignancy (p = 0.001). An inverse relationship between smoking and mortality was noted (p = 0.017) (Table 4).

**Table 4:** Statistics of data. Note: Normally distributed data are shown as mean ± SD (minimum-maximum), and non-normally distributed data as median (IQR 25-75). Student’s t-test was used to compare normally distributed data and Mann-Whitney U test was used to compare non-normally distributed data. IQR: interquartile range; HT: hypertension; DM: diabetes mellitus; CAD: coronary artery disease; CRF: chronic renal failure; CVD: cerebrovascular disease; SpO_2_: oxygen saturation; GGO: ground-glass opacity; IVC-AP: inferior vena cava anteroposterior diameter, IVC-Tr: inferior vena cava transverse diameter; m-PA: main pulmonary artery diameter; r-PA: right pulmonary artery diameter; l-PA: left pulmonary artery diameter

	All patients (n = 404)	Survival (n = 343)	Mortality (n = 61)	P-value
Age (years)	62 (IQR 49–72)	60 (IQR 47–70)	74 (IQR 68–81)	0.001
HT	203 (50.2%)	162 (40.1%)	41 (10.1%)	0.004
DM	114 (28.2%)	89 (22.0)	25 (6.2%)	0.016
CAD	71 (17.6%)	48 (11.9%)	23 (5.7%)	0.001
CRF	29 (7.2%)	22 (5.5%)	7 (1.7%)	0.158
CVD	11 (2.7%)	7 (1.7%)	4 (1.0%)	0.001
Malignancy	32 (7.9%)	20 (4.9%)	12 (3.0%)	0.001
Smoking	51 (12.6%)	49 (12.1%)	2 (0.5%)	0.017
Fever	36.5 (IQR 36.4–36.7)	36.5 (IQR 36.4–36.7)	36.5 (IQR 36.4–36.8)	0.356
Spo2	95 (IQR 92–98)	97 (IQR 93.5–98)	87 (IQR 80–91)	0.001
m-PA	26.7 (IQR 24.1–28.7)	26.2 (IQR 23.8–28.3)	28.5 (IQR 26.8–31.0)	0.001
r-PA	21.0 (IQR 18.5–23.3)	20.6 (IQR 18.0–22.8)	23.2 (IQR 20.8–25.4)	0.001
l-PA	20.4 (IQR 18.3–23.1)	19.9 (IQR 18.0–22.7)	23.0 (IQR 21.3–26.0)	0.001
IVC-Tr	31.0 (IQR 28.1–34.0)	30.9 (IQR 28.0-33.8)	31.9 (IQR 29.0–36.0)	0.045
IVC-AP	21.1 (IQR 19.0–23.9)	21.0 (IQR 18.8–23.7)	22.0 (IQR 19.2–25.5)	0.053
CT parenchyma score	3.0 (IQR 1.0–4.0)	2 (IQR 0–4.0)	5 (IQR 2.0–7.0)	0.001
GGO	285 (70.5%)	233 (57.6%)	52 (12.9%)	0.006
Consolidation	78 (19.3%)	66 (16.3%)	12 (3.0%)	0.937
Halo sign	15 (3.7%)	14 (3.5%)	1 (0.2%)	0.353
Reverse halo sign	27 (6.7%)	26 (6.5%)	1 (0.2%)	0.087
Pleural effusion	24 (5.9%)	14 (3.5%)	10 (2.4%)	0.001

The initial dataset was created using the lung parenchyma score, pulmonary artery diameters, and IVC dimensions. When models were run for mortality prediction, 94.9% accuracy was obtained with GB, 93.8% with XGBoost, and 93.4% with RF.

For the second dataset, thorax CT parenchymal findings were added to the initial dataset. In these models, 92.4% accuracy was achieved with GB, 90.8% with XGBoost, and 90.7% with NN. The decrease in accuracy was thought to be due to the noise created by the statistically limited information in the parenchymal findings. Based on this result, parenchymal findings were excluded from the third and fourth datasets.

The third dataset was reconstructed to include laboratory parameters in addition to the lung parenchyma score, pulmonary artery diameters, and IVC dimensions. Afterward, an accuracy rate of 98.4% was reached with GB, 96.8% with XGBoost, and 95.6% with RF.

Lastly, for the fourth dataset, the patient’s age, oxygen saturation, fever, and presence of comorbid diseases were added to the dataset. When the models were run, 97.1% accuracy was achieved with the GB model, 97.1% with RF, and 96.8% with XGBoost. All results including the validation and test results are summarized in Table 5.

**Table 5:** Comparison of mortality prediction models with different datasets. kNN: K-nearest neighbors; SVM: support vector machine; SGD: stochastic gradient descent; IVC: inferior vena cava; m-PA: main pulmonary artery diameter; r-PA: right pulmonary artery diameter; l-PA: left pulmonary artery diameter; SpO_2_: oxygen saturation

Models(validation/test results, %)	Dataset includes			
	CT parenchyma score, m-PA, r-PA, I-PA, and IVC diameters	CT parenchyma score, m-PA, r-PA, I-PA, IVC diameters, and parenchymal findings	CT parenchyma score, m-PA, r-PA, I-PA, IVC diameters, and laboratory parameters	CT parenchyma score, m-PA, r-PA, I-PA, IVC diameters, laboratory parameters, comorbid disease, age, fever, and SpO_2_
kNN	90.2/86.2	88.1/87.7	94.4/93.8	92.5/94.1
SVM	62.2/46.7	62.5/61.2	73.6/65.0	86.5/78.3
SGD	79.2/73.9	82.6/71.3	88.7/85.0	95.8/92.5
Random forest	92.1/93.4	91.7/87.8	95.3/95.6	97.5/97.1
Neural network	86.7/87.3	89.5/90.7	95.1/92.8	97.3/96.6
Naive Bayes	78.0/71.6	80.5/71.7	87.9/85.8	92.8/91.2
Logistic regression	79.2/75.6	82.9/73	88.3/86.5	94.6/92.6
AdaBoost	81.6/81.7	81.7/83.9	83.7/90.5	89.8/86.8
Gradient boosting	93.2/94.9	92.8/92.4	96.6/98.4	97.6/97.1
XGBoost	92.4/93.8	93.0/90.8	96.4/96.8	97.4/96.8

## Discussion

The rapid spread of the COVID-19 infection caused the intensive care units in hospitals to be filled and the inability to hospitalize patients in need. Predicting the mortality risk of patients in the emergency room quickly and effectively became crucial for health professionals during the pandemic.

Lung CT has been used extensively since the onset of the pandemic to show and diagnose abnormalities in the lung parenchyma. Despite its sensitivity of up to 90%, its specificity is low because the findings cannot be differentiated from other viral pneumonia agents [11]. However, its high sensitivity is beneficial in terms of showing the severity of the disease [12]. Different parenchymal scoring systems have been developed in the literature to quantify lung parenchymal involvement due to COVID-19 infection [13,14]. The general view is that mortality risk increases with the high parenchymal score, as in our study. There are systems in the literature that segment the involvement of the parenchyma with high accuracy using deep learning models [15]. These models can be used in place of parenchymal scoring and can increase the accuracy of mortality estimation.

While planning this study, we paid particular attention to the diameter of the pulmonary arteries and IVC. In autopsy studies performed on COVID-19 patients, diffuse alveolar damage in the lung as well as thrombosis in small and medium-sized pulmonary arteries were observed [16]. Thrombosis in the pulmonary arteries is thought to lead to increased resistance in the vascular bed and enlargement of the pulmonary arteries and right ventricle [17]. Therefore, the diameters of the main vascular structures may be a guide in predicting the outcome of COVID-19, and our results support this [18].

ML algorithms are extensively researched in the diagnosis and prognosis of COVID-19. In a meta-analysis of 33 studies, the average performance of ML models in mortality prediction was 0.93 for AUC [19]. In 2,924 patients with 152 features, including age, gender, comorbid diseases, vital signs at baseline, clinical symptoms, and laboratory tests, the GB model showed the best result with an AUC of 0.94 [20]. In another study based on similar features, AUC values ranging between 0.84 and 0.89 were obtained with the XGBoost model, which is an advanced version of the GB model [21]. Zakariaee et al. added CT parenchyma score to the dataset consisting of demographics, clinical data, comorbid diseases, and laboratory results [22]. In the study comparing kNN, multilayer perceptron, SVM, and J48 decision tree algorithms, an AUC of 0.97 was obtained with the kNN model, and it was observed that the addition of CT parenchyma score improved model performance. Another study compared model performances with similar datasets; J48, SVM, multilayer perceptron, k-NN, naive Bayes, LR, RF, and XGBoost models were compared and an AUC of 99.9% was obtained with the RF model [7]. The k-fold cross-validation technique was used in the study, but the validation results were not reported in terms of overfitting. In our study, we obtained similar results to the results obtained with demographic, clinical, and laboratory data in the literature with data obtained only from thorax CT. When six laboratory tests were added to these data, we obtained an above-average AUC of 0.98 with the GB model.

The effectiveness of the GB model in delivering reliable and valid results makes it a preferred choice for predictive modeling tasks, especially in complex and dynamic datasets where interpretability and adaptability are crucial. Li et al. obtained the highest accuracy with GB in their study for the prediction of mortality in intensive care unit patients with sepsis [23]. On the other hand, successful results have been obtained with the XGBoost, which is a specialized and optimized implementation of the GB model, in some studies conducted for the prediction of mortality in COVID-19 patients [24,25]. In our study, we obtained nearly similar results compared to GB in the XGBoost model. RF, another decision tree-based model, showed high performance in our study, and it appears to be the model with the best performance in some studies in the literature [26,27].

The main limitation of this study is that our data were obtained retrospectively from a single center. Moreover, there was a distribution imbalance between the data groups which was tried to be solved by the SMOTE method. These limitations can be resolved in future studies by creating more balanced datasets with multicenter studies. In addition, parenchyma scoring is done visually, which can be resolved by segmentation with a convolutional neural network.

## Conclusions

The mortality risk of patients can be predicted with high accuracy using the power of the GB model based on simple measurements from thorax CT and laboratory findings at admission.
